# Biofilm Resilience: Molecular Mechanisms Driving Antibiotic Resistance in Clinical Contexts

**DOI:** 10.3390/biology14020165

**Published:** 2025-02-06

**Authors:** Ahmad Almatroudi

**Affiliations:** Department of Medical Laboratories, College of Applied Medical Sciences, Qassim University, Buraydah 51452, Saudi Arabia; aamtrody@qu.edu.sa

**Keywords:** biofilm, EPS, antibiotic resistance, molecular mechanism, prevention

## Abstract

Healthcare-associated infections pose a significant global health challenge, negatively impacting patient outcomes and burdening healthcare systems. A major contributing factor to healthcare-associated infections is the formation of biofilms, structured microbial communities encased in a self-produced extracellular polymeric substance matrix. Biofilms are critical in disease etiology and antibiotic resistance, complicating treatment and infection control efforts. Their inherent resistance mechanisms enable them to withstand antibiotic therapies, leading to recurrent infections and increased morbidity. This review explores the development of biofilms and their dual roles in health and disease. It highlights the structural and protective functions of the EPS matrix, which shields microbial populations from immune responses and antimicrobial agents. Key molecular mechanisms of biofilm resistance, including restricted antibiotic penetration, persister cell dormancy, and genetic adaptations, are identified as significant barriers to effective management. Biofilms are implicated in various clinical contexts, including chronic wounds, medical device-associated infections, oral health complications, and surgical site infections. Their prevalence in hospital environments exacerbates infection control challenges and underscores the urgent need for innovative preventive and therapeutic strategies. This review evaluates cutting-edge approaches such as DNase-mediated biofilm disruption, RNAIII-inhibiting peptides, DNABII proteins, bacteriophage therapies, antimicrobial peptides, nanoparticle-based solutions, antimicrobial coatings, and antimicrobial lock therapies. It also examines critical challenges associated with biofilm-related healthcare-associated infections, including diagnostic difficulties, disinfectant resistance, and economic implications. This review emphasizes the need for a multidisciplinary approach and underscores the importance of understanding biofilm dynamics, their role in disease pathogenesis, and the advancements in therapeutic strategies to combat biofilm-associated infections effectively in clinical settings. These insights aim to enhance treatment outcomes and reduce the burden of biofilm-related diseases.

## Simple Summary

Biofilms are groups of microorganisms that stick together and form a protective layer, making them extremely difficult to eliminate with standard antibiotics. These biofilms often develop on medical devices, such as catheters and implants, or in wounds, causing long-lasting infections that are hard to treat and pose serious challenges in healthcare settings. This study investigates the structure of biofilms, how they protect bacteria from treatments, and their role in healthcare-associated infections. It highlights biofilms’ significant burden on healthcare systems, including prolonged hospital stays and increased treatment costs. To combat biofilm-related infections, the study explores innovative strategies such as nanoparticles that deliver targeted treatments, specialized enzymes that break down biofilms, and advanced coatings for medical devices that prevent biofilm formation. It also emphasizes the importance of early detection using advanced diagnostic tools, which can help tailor treatments to individual infections and improve outcomes. These advancements are vital for improving patient care, reducing infections, and creating safer healthcare environments. By addressing the challenges of biofilm-associated infections, this research contributes to developing better treatment options and more effective prevention strategies for patients and healthcare providers alike.

## 1. Introduction

Biofilms, organized microbial communities enveloped in a self-synthesized extracellular polymeric substance (EPS) matrix, significantly contribute to healthcare-associated infections (HAIs) and other microbial illnesses. EPS is a complex matrix synthesized by bacteria in biofilms, providing structural support and protection. EPS, consisting of polysaccharides, proteins, extracellular DNA (eDNA), and lipids, improves adhesion, sequesters nutrients, and protects bacteria from external pressures, such as antibiotics and immunological reactions. Its involvement in antibiotic resistance makes it a critical target for methods designed to break biofilms and address related illnesses [1,2,3].

The World Health Organization (WHO) predicts that in acute-care hospitals, 7% of patients in high-income countries and 15% in low- and middle-income countries would acquire at least one HAI during their hospital stay. Disturbingly, one in ten patients impacted by HAI do not survive, highlighting the urgent need for improved infection prevention and control measures [4]. In the United States, HAIs account for over 88,000 fatalities annually, with an estimated economic burden of USD 4.5 billion [5]. The yearly financial burden of HAIs in the United Kingdom is estimated at GBP 774 million, highlighting their significant impact on the healthcare system [6]. Biofilms are associated with around 65% of human microbial infections and 80% of chronic illnesses, highlighting their significant influence on healthcare systems [7,8,9].

Biofilm’s distinctive structure and adaptive mechanisms offer defense against antimicrobial agents, positioning them as significant contributors to antimicrobial resistance. Critical elements like bacterial cell density, quorum-sensing communication, and genetic adaptations, such as efflux pump activation and mutations, contribute to their resilience, making treatment efforts more challenging [10].

The relationship between biofilms and HAIs raises significant concerns. Biofilm-forming bacteria, including *Staphylococcus aureus*, *Coagulase-negative staphylococci* (CoNS), *Pseudomonas aeruginosa*, and *Acinetobacter baumannii*, exhibit resistance to antibiotics that can be as much as 1000 times greater than that of their planktonic counterparts [11,12,13]. This increased resistance extends the duration of infections and markedly elevates the likelihood of recurrence, especially in clinical settings.

In demanding settings like hospitals, microbes’ capacity to develop biofilms serves as a vital survival mechanism. Biofilms enable bacteria to withstand disinfectants, scarce nutrients, and antimicrobial agents, facilitating their prolonged survival on surfaces and devices [14,15]. Furthermore, their capacity to endure toxins and adjust to ecological disturbances amplifies their influence on healthcare systems, establishing them as a significant obstacle to efficient infection control [16,17].

**Figure 1:**
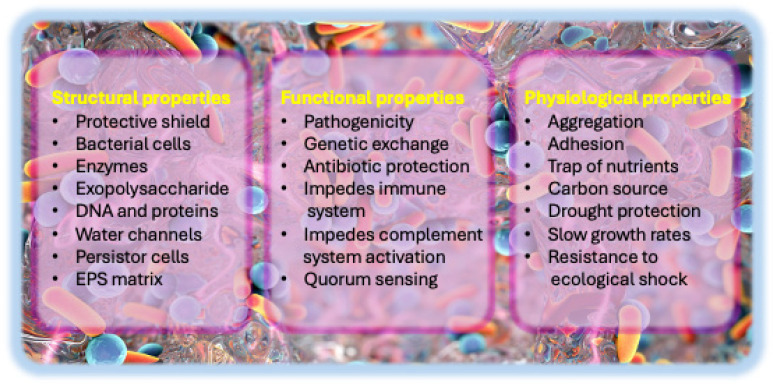
Different physicochemical and biological properties of biofilms.

To tackle the challenges presented by biofilms, it is essential to gain a more profound insight into their physicochemical and biological characteristics, as illustrated in [Figure 1]. The insights gained are crucial for formulating novel therapeutic approaches to disrupt biofilms, improve antibiotic effectiveness, and reduce their effects on healthcare.

Biofilms have long been recognized as a major contributor to HAIs, posing significant challenges due to their inherent resistance to antimicrobial treatments. Previous review articles have provided detailed insights into biofilm formation and resistance mechanisms. While these reviews have significantly advanced understanding of biofilm biology, they often focus predominantly on either the mechanistic underpinnings of biofilms, creating a gap between foundational science and translational applications. Furthermore, existing reviews have not fully addressed the economic burden or operational challenges of managing biofilm-associated infections nor provided a holistic analysis of emerging therapeutic and diagnostic advancements. This review aims to address these critical gaps by integrating recent advancements in therapeutic strategies, diagnostic technologies, and the economic aspects of biofilm management. While previous studies have emphasized conventional antimicrobial treatments, this work expands on innovative approaches such as nanoparticle-based therapies, enzymatic biofilm disruption, antimicrobial coatings, and other cutting-edge interventions. In parallel, it critically evaluates the clinical applicability and feasibility of advanced diagnostic tools, including biosensors, and molecular diagnostics, essential for early detection and tailored therapeutic decision-making in biofilm-associated HAIs. In addition, this work highlights the limitations of existing strategies and introduces novel solutions for the prevention, detection, and management of biofilm-associated infections.

## 2. Methods

A thorough literature review was conducted using databases such as Google Scholar, PubMed, and Scopus, focusing on articles published from 1 January 2018 to 31 December 2024. The search utilized keywords including biofilms, HAIs, bacterial adhesion, and microbial drug resistance. The articles that were included comprised experimental research, randomized controlled trials, reviews, and epidemiological studies. Articles were excluded based on the following criteria: editorials, theses, case reports, and abstract not in English.

## 3. Understanding Biofilms

### 3.1. Definition and Characteristics of Biofilms

**Figure 2:**
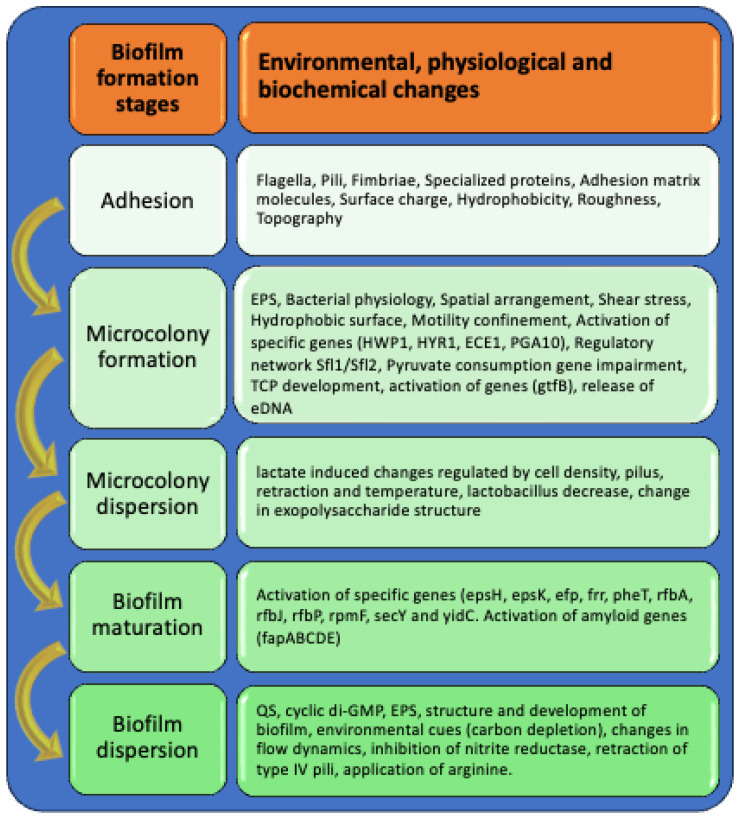
Role of different biomolecules in the life cycle stages of biofilm.

Biofilms are complex and organized microbial communities surrounded by an EPS. They play a crucial role in developing some diseases [18]. The creation of biofilms involves a series of stages, starting with reversible and irreversible attachment, proceeding to the production of EPS, and finally culminating in maturation [19] [Figure 2]. The biofilm matrix consists of eDNA, proteins, and polysaccharides, with its primary functions being to provide structural support and protect the microbial community from external factors [20].

Biofilms are known for their intricate architecture, organized development, and association with various human illnesses [18]. Their growth requires regulated physiological processes and cellular structure changes [21,22]. Biofilms are diverse and can facilitate genetic variation, enhancing their resilience and adaptability [23]. The multifaceted biofilm formation process involves various signaling pathways and transcription factors in microorganisms like *Candida albicans* and *Listeria monocytogenes* [24,25]. Additionally, they play a crucial role in the development of infections related to urinary catheters, periodontal disorders, and chronic rhinosinusitis [26,27,28]. *P. aeruginosa* is often present in biofilms associated with chronic wounds, including diabetic foot and venous leg ulcers. These biofilms hinder wound healing by inducing chronic inflammation and establishing a physical barrier to antibiotics and immune cells [29]. Biofilms produced by *S. aureus* on indwelling medical devices, including catheters, implants, and prosthetic joints, contribute significantly to device-associated infections. Owing to their significant resistance to antibiotics, these biofilms often need to be removed from devices [30].

### 3.2. Role of Biofilm Formation in Disease Pathogenesis

Biofilms significantly contribute to persistent infections as they attach to organic (tissues and teeth) and inorganic surfaces (such as medical implants and catheters) [31,32]. Once formed, biofilms are exceedingly challenging to eliminate, resulting in long-lasting and recurring infections. Biofilms are responsible for approximately 80% of difficult-to-treat, long-lasting infections, which presents a significant challenge in clinical treatment [7]. Infections that persist, such as equine recurrent uveitis, often involve biofilm production, which can impede treatment efforts [9].

Biofilm-associated organisms exhibit markedly increased antibiotic resistance compared to their planktonic counterparts. This enhanced resistance can be attributed to factors such as restricted drug penetration, scarcity of nutrients, and adaptive stress responses [10]. Moreover, pathogens gain multiple advantages from producing biofilms, including protection against the host’s immune system, resistance to antibiotics, and the recurrence of infections [33]. Biofilms can evade the host’s immune system through the EPS matrix, a physical obstacle for immune cells. Additionally, in some cases, biofilms can even incite immunological responses that result in damage to the surrounding tissues [34].

**Figure 3:**
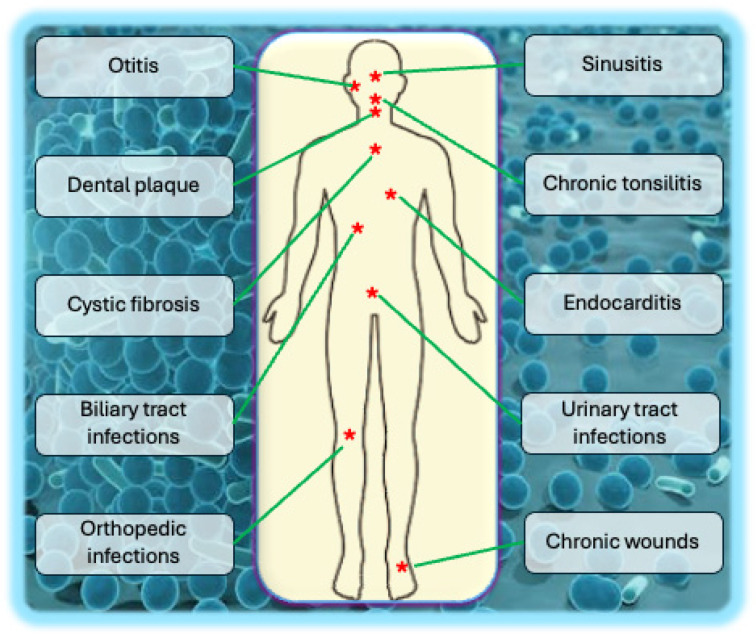
Pathogenic effects of different types of biofilms at different sites.

Notable examples of diseases linked to biofilms include chronic wound infections, where biofilms hinder the healing process; dental plaque causing tooth decay and gum disease; persistent lung infections in individuals with cystic fibrosis; and infections associated with medical implants such as catheters and prosthetic joints [35,36,37,38]. For instance, managing lung infections in cystic fibrosis is notably tricky because of the biofilm development pattern shown by pathogenic microorganisms. In contrast to planktonic bacteria, biofilms have a notable capacity to endure very high levels of antimicrobials, substantially reducing treatment effectiveness. Bacterial biofilms in the lungs of individuals with cystic fibrosis include clusters of microorganisms encased in EPS, all immersed in the dense sputum typical of the condition. This matrix promotes bacterial resistance and obstructs antibiotic penetration, contributing to chronic lung infections’ persistence [39,40]. The pathogenic effects of different types of biofilms at different sites are described in Figure 3.

### 3.3. Biofilm Formation

#### 3.3.1. Adhesion

The first step in the development of biofilms is adhesion, a complex process involving various structural and molecular components. Flagella, pili, fimbriae, and specialized proteins are appendages that facilitate this process, with each element serving a unique role in helping microbial cells attach to surfaces [41,42,43].

Flagella are essential for bacterial motility and play a critical role in chemotaxis, allowing bacteria to actively navigate toward favorable conditions and establish initial contact with surfaces. Chemotaxis enables microorganisms to move in response to chemical gradients, either toward beneficial stimuli or away from harmful ones [44]. Various biological systems control this survival mechanism. For example, in *E. coli*, chemotaxis is regulated by signaling arrays of chemotaxis protein paralogs, which coordinate signaling output [45]. Additionally, the *tonB* gene has been shown to influence bacterial movement, chemotaxis, attachment, and biofilm formation in *Pseudomonas plecoglossicida* [46]. Once bacteria reach a surface, alternative adhesion mechanisms reduce the functional significance of flagella in biofilm formation [41].

Fimbriae are critical for adhesion, particularly during the initial stages of biofilm formation. They enable bacteria to adhere to surfaces, other bacteria, or host cells, facilitating stable attachment [47,48]. Pili also contribute to adhesion and movement, with certain types, such as Type IV pili, playing a key role in “twitching motility”, which aids in disseminating and developing biofilms [49]. In addition to facilitating movement, pili are involved in intercellular adhesion within biofilms, contributing to the formation of intricate, layered structures. These structures enhance the ability of bacteria to effectively attach to and thrive on various surfaces [42].

Microbes that lack the ability to move use distinct methods for colonizing surfaces compared to their motile counterparts. These immobile bacteria rely heavily on surface chemicals and changes in the flow field around them to effectively adhere to surfaces. According to recent research, even slight modifications in the flow field are crucial for facilitating the adhesion of non-motile cells to adjacent surfaces [50]. Furthermore, outer sheath proteins have been proposed to play a significant role in colonizing microorganisms incapable of movement [51]. Pathogenic bacteria like *S. aureus*, for instance, can stick to epithelial surfaces by utilizing microbial surface components that recognize adhesive matrix molecules (MSCRAMMs). These components make the host more vulnerable to invasive infections [52].

The adhesion process is influenced by various factors, including the mechanical properties of the biofilm and the characteristics of the surface it adheres to. Biofilms possess the remarkable ability to modify the shape and structure of the surfaces they occupy through a process known as buckling adhesion, which can profoundly impact their interaction with the surface [53]. This dynamic interaction can also affect the physical environment.

The initial phases of biofilm formation are influenced by physicochemical aspects such as surface charge, hydrophobicity, roughness, topography, and chemistry. The collective impact of these variables affects bacterial adherence, thus shaping the early development of biofilms [54]. The physical characteristics of surfaces hold significant sway over bacterial adherence and the creation of biofilms. Surface nanoroughness may affect bacterial adherence, with superhydrophobic surfaces reducing bacterial adhesion [55]. Recent studies have confirmed that submicron topography on surfaces can significantly reduce bacterial adhesion and prevent biofilm formation [56]. These insights have practical implications, including the development of antimicrobial coatings to reduce biofilm formation. Hydrophilic coatings, like those with poly (ethylene glycol) (PEG), diminish microbial adherence by establishing a water-saturated surface that inhibits bacterial attachment [57,58]. Likewise, nanostructured surfaces engineered to reduce roughness may impede the mechanical adhesion of microorganisms. Progress in material science, including nanopatterned and self-cleaning surfaces, presents intriguing approaches to address biofilm-related illnesses in medical and industrial environments [59,60].

#### 3.3.2. Microcolony Formation

Following the initial adhesion of individual cells to a surface in this stage, there is a significant increase in cell multiplication and clustering. This leads to the formation of small groups of cells known as microcolonies. Microcolonies are characterized by the proximity of cells, which is crucial for subsequent phases of biofilm formation [61]. This proximity enables important intercellular communication and collaboration, creating conditions for a more structured community. This stage is marked by certain activities and structures that establish the groundwork for a fully developed biofilm, a sophisticated and durable collection of bacteria [62]. During the microcolony stage, bacteria transition from individual cell lifestyles to communal ones, marked by many cells and physical contact between them. This lifestyle shift can potentially influence their behavior [63].

During cell division, there is a notable uptick in the production EPS. These substances fortify microcolonies’ structural integrity, protect the cells against environmental stressors, and assist in retaining moisture and nutrients. The EPS matrix is a vital element of biofilms, contributing significantly to their remarkable resilience and stability [64,65].

During the microcolony stage, bacteria utilize quorum sensing (QS) as a chemical communication. By perceiving their density, cells can effectively coordinate group actions like gene expression changes [66]. Creating microcolonies involves activating specific genes that promote these clusters’ growth and pathogenic properties. In *C. albicans*, the development of microcolonies is significantly affected by the expression of genes such as *HWP1* (hyphal wall protein 1) and *HYR1* (hyphal regulator 1). *HWP1* is essential for adhesion and the formation of hyphal structures, which are vital for biofilm stability. Likewise, *HYR1* governs hyphal development and aids in the shift from yeast to hyphal growth, promoting microcolony aggregation and structural maturity. These mechanisms are crucial for establishing the thick, three-dimensional structure typical of *C. albicans* biofilms [67,68]. The regulatory network Sfl1/Sfl2 also governs the transcription of essential microcolony genes, impacting their development [61]. In *Vibrio cholerae*, gene expression responsible for the toxin-coregulated pilus (TCP) development is crucial for microcolony production [69].

As microcolonies expand, they give rise to distinct microenvironments due to differing concentrations of nutrients, oxygen, and waste products. Studies have proven that microcolonies exhibit regions with a neutral pH at the outer edge of the biofilm, acidic pockets within the microcolony, and low-pH zones at the junction between the microcolony and the surface it adheres to [70]. The different biomolecules play a role in the life cycle stages of biofilm [Figure 2].

#### 3.3.3. The Process of Maturing or Reaching Full Development

One crucial aspect of biofilm maturation is the development of water channels that function like a circulatory system. These channels efficiently transport nutrients and eliminate waste to ensure the viability of cells within the inner regions. These channels also contribute to the biofilm’s resilience by maintaining a harmonious environment within the structure [71,72].

Throughout the maturation process, a biofilm does not simply grow in size, it forms an intricate and advanced three-dimensional structure. The arrangement of this structure is meticulously organized, maximizing the biofilm’s effectiveness in utilizing resources and protecting itself from external dangers. The spatial organization inside the biofilm enables efficient resource allocation and confers a tactical benefit in defending against antagonistic chemicals, such as antibiotics or immunological reactions [73].

A dynamic alteration in gene expression distinguishes the maturation phase. The upregulation or downregulation of certain genes reflects the biofilm’s dynamic requirements. These genes are crucial in EPS production, stress response, nutrition acquisition, and cell–cell communication facilitation [74,75]. Differential gene expression plays a pivotal role in enabling biofilms to adapt and thrive under diverse environmental conditions. Recent studies have identified specific genes exhibiting increased activity during the maturation stage of biofilm development. For instance, in Bifidobacterium longum FGSZY16M3, genes such as *epsH*, *epsK*, *efp*, *frr*, *pheT*, *rfbA*, *rfbJ*, *rfbP*, *rpmF*, *secY*, and *yidC* were found to be significantly upregulated, contributing to polysaccharide biosynthesis and structural integrity of the biofilm matrix [76]. Similarly, the elevated expression of functional amyloid genes *fapABCDE* in fully matured biofilms of Pseudomonas fluorescens underscores their critical role in biofilm formation, facilitating adhesion and stability. Notably, the *fapC* mutant exhibited defects in forming mature biofilms, further emphasizing the importance of these genes in biofilm development [77,78]. These findings highlight the diverse genetic adaptations that support biofilm maturation and resilience.

The maturity period of biofilms varies across bacterial species and is affected by environmental conditions, including nutrition supply, temperature, and pH. Biofilm maturation often occurs over hours to days, depending upon the organism and ecological circumstances. In *P. aeruginosa*, biofilm maturation starts within 13 h under laboratory conditions, evolving into a well-organized and densely populated community by 24 to 48 h [79]. This phase entails the significant creation of EPS, which provides structural integrity and protection. Likewise, *E. coli* biofilms on urethral catheters achieve maturity within 24 h, underscoring the fast formation of biofilms in clinical environments [80,81].

#### 3.3.4. Dispersion

The dispersion phase is a vital part of the biofilm’s lifecycle, wherein cells that were formerly part of the community break apart and disperse to new locations [82,83].

Various environmental cues typically trigger this phase, including nutrient depletion, shifts in oxygen levels, waste accumulation, chemical signals and changes in flow dynamics. These changes are crucial in signaling bacteria within the biofilm to initiate the dispersion process [84,85]. As a result, the bacteria undergo significant genetic changes. Genes that maintain the biofilm structure decrease activity, while those that promote mobility and dissociation increase activity. This alteration in gene expression is vital in preparing the cells for detachment [86,87].

A crucial element of dispersion is the reduction in the production of EPS. As the synthesis of these structural and protective chemicals decreases, the biofilm matrix weakens, and bacteria generate specialized enzymes that aid in its degradation. This promotes the separation of cells from the biofilm [88]. For instance, dispersion B (DspB), an enzyme, has been demonstrated to facilitate the breakdown of poly-N-acetylglucosamine (PNAG), a significant polysaccharide found in biofilms produced by different bacteria that create biofilms [89]. In numerous Gram-positive bacteria, such as *S. aureus*, biofilm dispersal is governed by the Agr QS system, which monitors bacterial cell density. Upon reaching a specific threshold of bacterial population, the Agr system initiates the synthesis of Phenol-Soluble Modulins (PSMs). These diminutive, amphipathic peptides exhibit cytotoxic properties and facilitate the disintegration of the biofilm matrix, hence enhancing the liberation of bacteria from the biofilm into the surrounding milieu [90,91].

#### 3.3.5. Influence of External Factors on Biofilm Formation

The conversion of planktonic microbes into biofilm-forming, adhering states is a meticulously controlled process affected by several external influences. These environmental stimuli serve as signals, prompting bacteria to form biofilm as a survival mechanism. Critical parameters like pH, temperature, and nutrient availability significantly influence this transition, affecting the behavior and resilience of biofilm-forming microbes.

- pH and Biofilm Formation

The pH levels in their surroundings significantly impact the ability of bacteria to produce biofilms. Depending on the pH levels, various types of bacteria display different capacities for creating biofilms. According to Mathlouthi et al. (2018), *E. coli* strains produce more biofilms when the medium pH is 7.4 [92]. Similarly, a study found that *Salmonella enterica* serotype Kentucky forms more biofilms at higher pH levels (7.0 and 8.0) than lower ones (4.0 to 6.0) [93].

- Temperature Effects

Temperature is a significant component influencing biofilm development. The optimal temperatures for biofilm formation differ across species. Studies have shown that different types of bacteria respond differently to temperature when it comes to the formation and growth of biofilms. For instance, *E. coli* was found to form biofilms most effectively at 25 °C, while it struggled at 37 °C [92]. Additionally, research by Chaumet et al. revealed that exposure to 26 °C for three days led to increased production of polysaccharides compared to 10 °C [94]. Furthermore, Ghazay and Mamdouh’s research suggested that the optimal temperature for *Klebsiella Pneumonia* biofilm growth is around 40 °C, as this was when the highest levels of biofilm formation were observed [95]. Increased or decreased temperatures may induce stress in microbial cells, promoting biofilm formation as a defensive mechanism against thermal stressors.

- Nutrient Availability

Nutrient availability serves as a primary catalyst for biofilm formation. Bacteria often demonstrate increased metabolic activity in nutrient-dense settings, promoting fast biofilm development. Nutrient deprivation may trigger biofilm development as a survival strategy, enabling bacteria to preserve resources and endure unfavorable circumstances [96,97]. *Salmonella* biofilms are more prone to formation in nutrient-deficient conditions, where adherence confers a competitive edge in resource acquisition [98]. Another study demonstrated that *P. aeruginosa* exhibited a 4.6-fold increase in biofilm formation under nutrient-limited conditions compared to nutrient-rich environments [97]. 

## 4. The Role of the Extracellular Polymeric Substance in Biofilm Formation and Function

EPS constitutes the structural basis of biofilms, significantly contributing to their unique three-dimensional architecture. Research indicates a dual role of EPS in biofilm formation. EPS promotes bacterial migration and plays a role in biofilm dispersion, thereby influencing the mobility of surface biofilms [99]. Secondly, EPS improves bacterial adhesion to surfaces, a critical process in the early stages of biofilm formation and its ongoing stability [100]. EPS functions as a biological adhesive, facilitating the adhesion of bacterial cells, providing mechanical support, and enhancing resistance to shear forces and physical disruptions [1]. Interactions between EPS and eDNA are essential for the spatial organization of biofilms, especially during their later developmental stages [101].

EPS safeguards biofilm cells against environmental stressors. The matrix is a barrier to ultraviolet (UV) radiation by reducing light penetration, thereby protecting bacterial DNA from UV-induced damage [102,103]. Additionally, EPS inhibits dehydration by sustaining elevated hydration levels, fostering a moist internal environment essential for microbial survival in low moisture or nutrient scarcity [104,105]. The hydration property is essential for maintaining the internal stability of the biofilm, particularly in adverse environmental conditions.

The EPS matrix enhances the biofilm’s resilience to extreme environmental fluctuations, such as temperature variations and changes in pH. EPS’s adaptability enables it to shield microbes from immune responses and restrict antibiotic penetration, thereby contributing to extended inflammation and resistance to standard antibiotic treatments. This resilience notably complicates the management of infections [2,3,106].

The conservation and distribution of nutrients within biofilms are significantly affected by EPS. The matrix captures and stores essential nutrients and growth factors, ensuring bacterial cells possess sufficient resources for survival and growth [107]. EPS contributes to nutrient cycling in periphytic biofilms by promoting the accumulation of nitrogen and phosphorus and enhancing organic carbon content [108]. These functionalities are particularly crucial in practical scenarios, such as enhancing biofilm resistance on medical implants, which may lead to chronic infections and complicate therapy [109,110].

**Figure 4:**
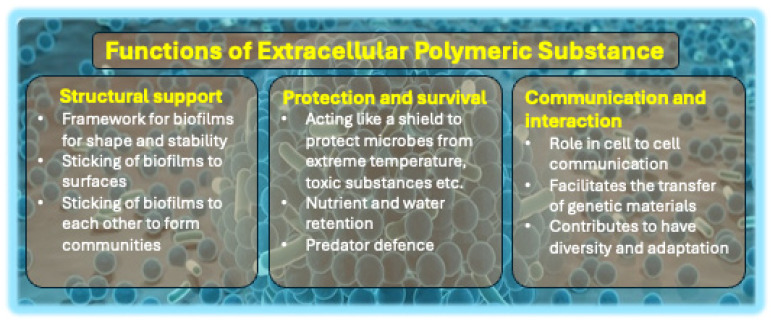
Different functions of EPS in biofilm structure, function, and integrity.

The compact structure of the EPS matrix facilitates close proximity among bacterial cells, thereby enhancing opportunities for genetic exchange. This proximity is essential for horizontal gene transfer, facilitating the dissemination of antimicrobial resistance genes and thereby increasing the adaptability and resilience of biofilm communities [111,112]. Different functions of EPS in biofilm structure, function, and interaction are presented in Figure 4.

## 5. The Molecular Mechanisms Behind the Resistance of Biofilms to Antimicrobial Agents

Studies have demonstrated the link between biofilm formation and the rise in antibiotic resistance in microbial cells. The regulation of biofilm production is governed by particular genes that influence the expression of antibiotic resistance, underscoring the genetic foundation of this occurrence [12]. Furthermore, bacterial cells that separate from biofilms maintain their antibiotic resistance characteristics, suggesting that this resistance continues to exist even when the cells shift into a planktonic form [113].

The remarkable characteristic of bacteria within biofilms is their capacity to withstand antimicrobial agents, which is higher than that of free-floating bacteria. This increased resistance significantly diminishes their vulnerability to antibiotics, posing substantial challenges in treatment [114,115]. The structure of biofilms serves as a physical barrier against antimicrobial agents, while the QS process—bacterial communication within the biofilm—controls genes related to defense and survival, thereby increasing resistance [116].

Biofilms exhibit a notable capacity for rapid antibiotic adaptation, even when exposed to low concentrations. This illustrates the significant selective pressure imposed by antimicrobial agents, propelling the rapid development of resistance mechanisms. This level of adaptability presents a considerable challenge in clinical treatments [117]. Moreover, the biofilm matrix, rich in proteins and eDNA, enhances resistance by adhering to antibiotics and diminishing their effectiveness. This composition protects bacterial cells and reduces the efficacy of antibiotics specifically formulated with anti-biofilm characteristics [118].

Tackling the significant challenge of antibiotic resistance linked to biofilms is essential for enhancing treatment approaches, especially in the fight against stubborn infections that resist standard therapies.

### 5.1. Physical Barrier of the Biofilm Matrix

The biofilm matrix acts as an essential structural and protective framework, playing a significant role in antibiotic resistance. Research indicates that the matrix limits the diffusion and penetration of antibiotics, thereby protecting bacterial cells within the biofilm from therapeutic agents [119,120]. The restricted permeability serves as a key contributor to the resistance seen in infections linked to biofilms. Antibiotics frequently struggle to penetrate bacterial cells situated beyond the outermost layer of the biofilm, which diminishes their effectiveness and enables the safeguarded inner populations to endure and continue existing [121].

The biofilm matrix serves as a physical barrier and triggers physiological changes in bacteria that contribute to increased resistance. For example, the matrix can react to sublethal levels of antibiotics by adjusting its synthesis. This adaptive response enhances the biofilm structure and promotes the rapid evolution of resistance mechanisms within the bacterial community [122].

Recent studies have highlighted the significance of mechanical signals in the three-dimensional microenvironment of biofilms. For instance, the rigidity of EPS matrix has been shown to affect the biofilm’s capacity to endure antibiotics. The mechanical properties could improve the biofilm’s resilience, allowing bacterial populations to withstand external stresses, such as antimicrobial treatments, more effectively [64].

### 5.2. Diminished Rate of Growth and Decreased Metabolic Activity

Bacterial biofilm cells’ diminished growth rate and modified metabolic functions play a crucial role in their heightened antibiotic resistance. The biofilm matrix presents a complex landscape where nutrient and oxygen levels fluctuate significantly, leading to unique metabolic conditions for each cell. The presence of these variations facilitates antibiotic tolerance and persistence, which permits specific subpopulations of bacteria to endure antimicrobial treatments [123,124]. The restricted availability of nutrients and reduced metabolic activity commonly observed in bacteria associated with biofilms enhance resistance mechanisms, significantly complicating biofilm infection eradication [12,125].

Comparative studies reveal significant disparities in microbial growth rates between bacteria associated with biofilms and those in a planktonic state. Studies indicate that bacteria within biofilms exhibit a growth rate of roughly one-third that of planktonic cells during the exponential growth phase. The diminished growth rate noted in *P. aeruginosa* biofilms aligns with observations in multiple bacterial species, highlighting the slower replication of cells associated with biofilms [126]. The diminished growth observed, particularly within the deeper layers of the biofilm, compromises the effectiveness of numerous antibiotics, which are typically formulated to target cells that are actively dividing.

Within biofilms, bacterial cells demonstrate notable metabolic diversity, especially when comparing the inner and outer layers of the biofilm architecture. Cells situated in the center of the biofilm encounter restricted oxygen levels, leading to decreased growth rates and lowered metabolic activity. This metabolic inactivity makes these cells more resistant to antibiotics, focusing on active cellular functions. Cells at the biofilm surface benefit from enhanced access to oxygen and nutrients, facilitating increased metabolic activity and higher replication rates [127].

Genetic studies have clarified the connection between reduced growth rates and antibiotic resistance in biofilm-associated bacteria. For example, in *Proteus mirabilis*, the downregulation of specific genes, including *pstS*, *sodB*, and *fumC*, is closely linked to slower growth and diminished metabolic activity. The alterations facilitate biofilm development, establishing a safeguarding environment that improves bacterial resilience to antimicrobial therapies. This genetic and metabolic adaptation is directly linked to heightened antibiotic resistance, underscoring biofilm communities’ resilience and capacity to circumvent therapeutic interventions [128].

### 5.3. Genetic Recombination and Genetic Variation Through Changes in DNA

The rise of antibiotic resistance in biofilms is intricately linked to genetic recombination and DNA polymorphism within these intricate microbial communities. Biofilms facilitate horizontal gene transfer because of their elevated cell density, genetic competency, and the presence of mobile genetic elements, including plasmids and transposons [129]. Recent studies have shown that biofilms can aid in the transfer of MDR plasmids, emphasizing the possibility of genetic exchange among bacterial populations. A trade-off between horizontal and vertical transmission of plasmid transfer genes has been noted, highlighting the dynamic interplay of evolutionary strategies within biofilms [130]. Genetic adaptation, influenced by mutations and recombination, serves as a fundamental aspect of biofilm resilience, allowing bacterial populations to flourish in challenging environments [131].

The presence of genetic variation in biofilms significantly influences antibiotic resistance, the process of biofilm formation, and the potential for pathogenicity. Experimental studies on pneumococcal biofilms indicate significant rates of gene transfer, implying that recombination events frequently occur in these densely populated environments [132]. The close proximity of cells in biofilms increases the chances of genetic exchange, facilitating the persistence and spread of resistance genes over time [133]. This genetic plasticity enhances biofilms’ defenses against antibiotics and propels the evolution of increasingly virulent and resistant bacterial strains [134].

Specific bacterial species, including *A. baumannii*, utilize biofilms as concentrated areas for the exchange of genetic information. This environment promotes the swift spread and development of resistance genes, exacerbating the medical challenges associated with biofilm-related infections [135]. The intrinsic phenotypic tolerance of biofilms to antibiotics, along with their ability to transfer resistance genes, highlights the significance of biofilms as reservoirs and amplifiers of antibiotic resistance [136].

### 5.4. Stress Responses and Adaptive Resistance

The capacity of bacteria to endure extreme conditions and resist antimicrobial agents is intricately linked to the stress responses and adaptive resistance strategies demonstrated within biofilms. Forming biofilms exemplifies a complex adaptive mechanism in response to various environmental stressors, such as physical pressures, chemical agents like hypochlorous acid, and antibiotic exposure [126,137,138,139]. The ability to adapt is crucial for biofilms’ resilience, allowing them to survive in harsh conditions that would usually hinder or eradicate planktonic bacterial cells.

Biofilms utilize various strategies to adjust to stress and improve their resilience. These encompass structural modifications, physiological adaptations, and the emergence of genetic resistance that enhance their survival in challenging environments. For example, biofilm-associated bacteria can alter their EPS matrix, modifying its viscoelastic characteristics to reduce the impact of mechanical and chemical stresses [140,141,142]. Furthermore, biofilms have the capability to trigger particular stress responses, including oxidative stress pathways and the SOS response, upon exposure to antibiotics. The pathways induced by stress lead to genetic alterations and repair processes that strengthen resistance, thereby improving the survival of biofilm communities [126].

The occurrence of stress-inducing agents, including triclosan and aminoglycosides, can initiate the emergence of adaptive resistance within biofilms. This resistance encompasses physiological alterations that enable bacteria to endure concentrations of antibiotics that would typically be lethal, underscoring the adaptive strategies for survival within biofilms [143]. Additionally, biofilms have demonstrated the ability to facilitate the development of resistance by encouraging genetic alterations, such as mutations and horizontal gene transfer, in response to stress conditions. These adaptations allow biofilm-associated bacteria to circumvent therapeutic interventions and establish enduring infections [144].

### 5.5. Quorum Sensing Systems and Their Regulation of Efflux Pumps

Many molecular processes regulate resistance associated with biofilms, notably QS and essential efflux pumps. QS is a cell-to-cell communication mechanism that employs signaling molecules, including acyl-homoserine lactones (AHLs) in Gram-negative bacteria and oligopeptides in Gram-positive bacteria, to synchronize bacterial action [145,146,147]. This mechanism allows biofilm bacteria to detect their population density and collectively modulate gene expression, including genes related to antimicrobial resistance and biofilm development [148].

The QS systems Las, Rhl, and Pqs in *P. aeruginosa* govern the expression of several efflux pumps, such as MexAB-OprM, MexEF-OprN, and MexCD-OprJ. These efflux pumps actively discharge diverse antibiotics, including β-lactams, fluoroquinolones, and aminoglycosides, facilitating MDR. The Las and Rhl systems govern the synthesis of signaling molecules such AHLs, which subsequently enhance the expression of efflux pump genes, augmenting the bacterium’s resilience against antimicrobial threats [149,150]. The Pqs system, using 2-heptyl-3-hydroxy-4-quinolone (PQS) signaling, has been associated with the augmentation of efflux pump activity, further facilitating antibiotic tolerance in biofilms [149].

In Gram-positive bacteria like *S. aureus*, the accessory gene regulator (agr) QS system regulates resistance mechanisms. The agr system regulates the NorA efflux pump, which expels fluoroquinolones and other hydrophobic substances. The QS-regulated synthesis of virulence factors and biofilm matrix constituents often correlates with heightened efflux pump activity, establishing a synergistic barrier to antimicrobial drugs [151,152].

Efflux pumps react to QS signals and affect QS signaling itself. In *P. aeruginosa*, efflux pumps like MexAB-OprM influence the internal levels of QS signaling molecules such as AHLs, establishing a feedback loop. Research has shown that the overexpression or chemical induction of the MexCD-OprJ efflux pump in *P. aeruginosa* can attenuate QS signaling [153,154]. This interaction guarantees that QS signals are maintained at appropriate quantities to coordinate collective bacterial activities successfully.

### 5.6. Persister Cells

Persister cells, a distinct group of dormant and highly antibiotic-tolerant bacterial cells, are essential in understanding the resistance of biofilms to antimicrobial treatments. The specialized cells found within biofilms have been recognized as a key factor in biofilm resistance and play a significant role in the recurrence of bacterial biofilms in medical and industrial settings [155]. In contrast to genetically resistant cells, persister cells exhibit a temporary form of resistance that arises not from genetic mutations but from their metabolic inactivity and dormancy, allowing them to avoid the effects of antibiotics [156]. Persister cells have been observed in a diverse array of bacterial and fungal species, such as *P. aeruginosa*, *E. coli*, *S. aureus*, *C. albicans*, *A. baumannii*, and *B. cereus* [12,157,158,159,160].

Studies indicate that persister cells exist in multiple bacterial species, especially in biofilms. In biofilms of *P. aeruginosa*, specific clusters of persister cells are found in the deepest layers, where oxygen and nutrients are scarce [157]. The association of these cells with mechanisms like the suppression of protein translation, induction of dormancy, and development of antibiotic tolerance highlights their role in surviving amid antimicrobial agents [12].

The presence of persister cells in biofilms rises markedly when they are subjected to elevated levels of antibiotics. Research has shown that biofilms contain more persister cells when exposed to antibiotic concentrations that significantly surpass the minimum inhibitory concentration needed to eradicate planktonic cells [161]. This phenomenon highlights the significance of persister cells in maintaining biofilm viability, even in the face of rigorous antimicrobial treatments.

The existence of persister cells is well-established. However, complex molecular processes that require more investigation regulate their creation and reactivation. A primary mechanism involves toxin–antitoxin systems, as shown by the hipBA module in *E. coli*. In this approach, the HipA toxin phosphorylates target proteins, blocking critical cellular activities such as translation and inducing a dormant state in cells. This dormancy offers protection against antibiotics that depend on active cellular processes for effectiveness [162,163]. Moreover, the stringent response, governed by (p)ppGpp signaling, is essential for persister cell production since it modulates bacterial metabolism under stress conditions. This communication reduces growth rates and energy expenditure, facilitating the shift into a metabolically dormant, antibiotic-tolerant condition [164,165]. Persister cells resume activity when external stressors, such as antibiotics, are removed. Reactivation is often linked to metabolic pathways, with energy generation as a key trigger for exiting dormancy [166,167]. Mechanisms and illustrations of biofilm resistance to antimicrobial agents [Table 1].

## 6. Biofilms and Healthcare-Associated Infections

### 6.1. Biofilms in Hospital Environments: A Persistent Challenge

Biofilms on hospital surfaces represent a significant issue due to their essential involvement in HAIs. Biofilms are surrounded by a protective EPS, which increases their survival and resistance to disinfection efforts. Biofilms are commonly found on moist and dry surfaces in diverse hospital environments, including medical equipment, furniture, and high-contact areas [169,170]. Moist biofilms, commonly observed in sinks and drains, are well-documented; however, the emergence of dry surface biofilms (DSBs) presents a significant challenge in infection control. Research demonstrates that approximately 95% of sampled hospital surfaces contain DSBs, underscoring their prevalence and persistence in healthcare settings [171].

DSBs pose distinct challenges, challenging the traditional notion that low-moisture environments are inhospitable to microbial survival. The EPS matrix facilitates the retention of localized moisture and nutrients by microorganisms, thereby supporting their survival on surfaces such as bed rails, tables, electronic devices, and door handles. This adaptation enables pathogens to survive for prolonged periods—weeks or months—on dry surfaces, even in adverse environmental conditions [172,173].

Biofilms, especially DSBs, present unique infection prevention and control challenges. Biofilms act as reservoirs for pathogens that may be transmitted via direct or indirect contact with patients, healthcare personnel, and medical devices [174]. Research indicates that *S. aureus* and *Clostridioides difficile* can contaminate gloves, hands, and clothing, thus promoting the transmission of infections in healthcare settings [175,176]. In contrast to moist biofilms, which are generally localized in specific environments such as plumbing systems, DSBs exhibit a broader distribution, often present on high-touch surfaces, thereby enhancing their potential for cross-contamination [177].

The significant role of biofilms, especially on dry surfaces, in exacerbating antimicrobial resistance (AMR) is a matter of considerable concern. The biofilm matrix functions as a physical barrier, limiting the penetration of antimicrobial agents and establishing a microenvironment conducive to the horizontal transfer of resistance genes. This protected environment facilitates the survival and proliferation of MDR and XDR pathogens, significantly complicating infection prevention and treatment strategies [123,178]. *A. baumannii* and *P. aeruginosa*, known for their biofilm-forming capabilities, are commonly found in hospital environments and are often associated with outbreaks of HAIs [179,180]. The ability to form biofilms on dry surfaces enhances resilience and increases the risk of antimicrobial resistance dissemination.

The microbial diversity in hospital biofilms is extensive, including bacteria and fungi. Pathogens, including, MDR ESKAPEE; *A. baumannii*, *K. pneumoniae*, *Enterobacter* species, *MRSA*, *E. coli*, *P. aeruginosa*, and *Enterococcus faecalis* and *faecium*, are frequently detected in biofilms on dry hospital surfaces [180,181,182,183]. *MRSA* demonstrates a notable capacity for survival on low-moisture surfaces, including bed rails and hospital furniture, where its biofilm state protects against desiccation and chemical disinfectants [184,185]. MDR *P. aeruginosa* and *E. coli* can form biofilms on dry surfaces, even though they typically prefer moist environments [186,187].

Fungal pathogens such as *C. albicans* and *Candida auris* contribute to biofilm formation on hospital surfaces. *C. auris* is associated with notable healthcare-related outbreaks, attributed to its capacity to form resilient biofilms, endure on hospital surfaces, and resist cleaning agents [188,189]. Fungal biofilms pose significant risks for immunocompromised patients, potentially resulting in severe systemic infections. Genetically identical *Candida parapsilosis* isolates are linked to outbreaks due to their capacity to form persistent biofilms [190].

The persistence of biofilms on dry surfaces complicates disinfection protocols. Numerous widely utilized disinfectants, including sodium hypochlorite, exhibit difficulty in effectively penetrating the biofilm matrix, resulting in the persistence of viable microorganisms that can recolonize surfaces [191]. This limitation underscores the necessity for novel cleaning and disinfection strategies.

### 6.2. Medical Device Infections

Developing bacterial biofilms on implantable medical devices presents a significant challenge in contemporary healthcare, greatly influencing device-related infections. Biofilms can form on various medical devices such as catheters, sutures, orthopedic implants, heart valves, and vascular grafts, significantly contributing to HAIs [15,192,193]. Upon establishment, biofilms provide a protective environment for bacterial communities by surrounding them with EPS matrix. This matrix improves adhesion to device surfaces and protects bacteria from immune defenses, making biofilm-associated infections particularly resistant to standard treatments [194,195].

Infections linked to biofilms present significant clinical challenges, leading to consequences that go beyond localized areas and can result in systemic, potentially life-threatening conditions. For example, biofilms on medical devices are associated with bloodstream infections stemming from central venous catheters, prosthetic joint infections, and infective endocarditis affecting heart valves. The recurrent nature of these infections can be attributed to the protective characteristics of the biofilm, allowing bacterial cells to persist in a dormant state, thereby evading immune detection and standard antimicrobial treatments. In severe instances, infections associated with biofilms can advance to sepsis—a widespread inflammatory reaction that carries a considerable mortality risk if not addressed swiftly [196,197,198].

Eliminating infections linked to biofilms presents significant challenges because of the natural resistance mechanisms present within biofilm structures. The EPS matrix obstructs the penetration of antibiotics while simultaneously prompting a phenotypic shift in bacterial cells, thereby diminishing their vulnerability to conventional treatments. Inactive bacterial cells embedded in the biofilm can endure extended antibiotic treatments, frequently resulting in device removal and replacement being the sole feasible treatment alternative [194,195].

The economic cost of biofilm-associated infections is substantial, placing a significant financial strain on global healthcare systems. Central venous catheter-related infections, heavily influenced by biofilm formation, are estimated to cost USD 11.5 billion globally. Similarly, catheter-associated urinary tract infections, affecting approximately 150 million individuals annually, contribute an additional USD 1 billion in healthcare expenses worldwide. These figures highlight the economic burden of managing biofilm-associated complications in catheter use [199].

Prosthetic joint infections represent another costly challenge driven by biofilm formation. The presence of biofilms on prosthetic surfaces often leads to antibiotic treatment failure, leaving surgery as one of the few viable options. Revision surgeries required to address biofilm-associated prosthetic joint infections contribute a staggering USD 7849 million annually to the global healthcare burden [199,200].

Biosensors provide an advanced solution for the early detection of biofilm formation on medical devices, including catheters, implants, and prosthetic joints. These diagnostic instruments may identify biofilm formation in its first phases, often before the manifestation of obvious infection indicators. Integrated with devices, biosensors use technologies such as electrochemical and piezoelectric transducers to detect changes in electrical signals or mass, signifying the existence of biofilms [201,202]. These sensors exhibit heightened sensitivity to biofilm-specific indicators, such as QS molecules and microbial metabolites, facilitating accurate and timely detection [203,204].

### 6.3. Chronic Wounds

Forming biofilms is a critical characteristic of chronic wounds, playing a substantial role in their persistence and negatively impacting healing outcomes [205]. In contrast to acute wounds, chronic wounds demonstrate an increased vulnerability to biofilm formation, with research indicating that biofilms are found in around 60% of chronic wounds [206]. The immune system typically effectively manages infections in acute wounds, promoting routine wound healing. Chronic wounds exhibit an imbalanced immune environment that facilitates the formation and proliferation of biofilms [205].

The EPS shield facilitates microbial persistence, sustaining colonization and resulting in chronic inflammation that interferes with wound-healing [207]. This persistent inflammatory condition creates a self-perpetuating cycle in which the growth of biofilms worsens immune dysfunction, thereby prolonging or preventing the healing of wounds [205]. Confronting the issue of biofilms in chronic wounds is crucial for enhancing treatment approaches and facilitating efficient wound care.

The intricacy of addressing biofilms is amplified in chronic wounds that often include polymicrobial biofilms, consisting of many bacterial and fungus species. These polymicrobial communities engage in synergistic interactions, increasing their collective resistance to medicines and complicating treatment approaches [208]. This enduring resistance hinders the successful elimination of biofilms, leading to chronic infections that extend inflammation and obstruct the restoration of normal tissue function. Addressing the complex resistance mechanisms of biofilms is essential for creating more effective treatment strategies in chronic wound care.

Biofilms are essential in the development and persistence of various chronic wound types, including diabetic foot ulcers, venous leg ulcers, pressure ulcers, and surgical wound infections. Each wound type presents distinct pathological conditions that impact biofilm formation and stability [209,210,211,212]. For instance, diabetic foot ulcers are associated with high glucose levels that promote bacterial growth and the formation of biofilms [213]. In the case of venous leg ulcers, chronic venous insufficiency creates an inflammatory and nutrient-rich environment, which also encourages biofilm development [214,215]. Once biofilms are established, they pose significant challenges to treatment. The EPS matrix acts as a barrier, shielding the infection from phagocytosis by immune cells and impeding the diffusion of antibiotics, which diminishes the efficacy of standard therapeutic interventions [216].

In chronic wound management, biosensors provide a revolutionary method for the real-time detection and monitoring of biofilms. These sensors are often integrated into wound dressings that treat and safeguard the wound while offering diagnostic functionalities. These biosensors provide early management by identifying particular biomarkers associated with wound infection. Frequently seen biomarkers include pH and uric acid, which serve as significant indications of illness advancement [217,218]. Moreover, a wider array of indicators, including lactic acid, oxygenation levels, inflammatory mediators, bacterial metabolites, and the presence of bacteria, may be detected by wearable biosensors [218,219]. These non-invasive tests enable doctors to make prompt and precise judgments, reducing the risk of biofilm-associated illnesses and facilitating expedited recovery. Integrating sophisticated detecting technologies into wound care and biosensors improves patient outcomes and clinical management efficiency.

### 6.4. Dental Health

In dental health, biofilms mostly appear as plaque, a complex microbial formation that develops on tooth surfaces. The development of dental plaque starts with the attachment of the first colonizing bacteria to the pellicle layer, a proteinaceous film on the tooth surface. As the biofilm develops, it becomes a highly organized microbial community encased in EPS matrix. This matrix provides structural integrity and protection, making bacterial colonies resistant to mechanical removal. Thus, managing biofilms continues to be a significant issue in dental hygiene [220,221,222].

The adverse effects of dental biofilms are most apparent in their contribution to developing dental caries and periodontal disorders. Dental caries arise from the metabolic processes of acidogenic bacteria, especially *S. mutans*. These bacteria digest dietary carbohydrates, producing lactic acid as a byproduct. The localized acidification in the biofilm environment decreases pH, resulting in the demineralization of dental enamel. This process triggers cavity development, advancing as enamel integrity deteriorates, allowing bacterial infiltration into more profound dental components, including the dentin and pulp. This development intensifies structural damage and elevates the likelihood of infection [223,224].

Periodontal disorders, including gingivitis and periodontitis, are intimately associated with biofilm formation at the gingival edge. Gingivitis, the preliminary phase of periodontal disease, is characterized by gum inflammation induced by the immune response to biofilm-related bacteria. If left untreated, gingivitis may advance to periodontitis, a more serious disorder characterized by the subgingival infiltration of biofilms. This advanced stage leads to the degradation of the periodontal ligament and alveolar bone, resulting in persistent inflammation, tissue degeneration, and tooth loss [225,226].

Biofilms provide considerable obstacles in dental implantology. The creation of biofilm on dental implants may result in peri-implantitis, a severe inflammatory illness linked to implant failure. Biofilms on implants provoke a persistent immunological response that, if unregulated, leads to the gradual deterioration of adjacent soft tissues and alveolar bone. This degradation undermines the structural integrity of the implant, eventually leading to its collapse. These problems underscore the need for appropriate biofilm control solutions in implant dentistry to improve long-term results and avert complications such as peri-implantitis [227,228].

### 6.5. Surgical Site Infections

Surgical site infections (SSIs), a prevalent type of HAI, result in extended hospitalizations, the necessity for additional surgical procedures, and prolonged antimicrobial treatments, thereby markedly elevating patient morbidity and mortality rates [229,230]. SSIs pose a considerable challenge in healthcare, with biofilms significantly contributing to their development and persistence [231]. SSIs arise post-surgery at the incision site or surrounding tissues. They impact roughly 0.5% to 3% of surgical patients [232,233].

The European Centre for Disease Prevention and Control reported that SSIs constituted up to 18.4% of HAIs documented in Europe from 2016 to 2017 [234]. At that time, SSIs represented 20% of all HAIs in the United States [235]. In addition to their common occurrence, SSIs are linked to substantial clinical outcomes, notably a markedly elevated risk of mortality. Patients with SSIs exhibit a mortality risk that is 2 to 11 times greater than that of patients without these infections. Approximately 75% of deaths associated with SSIs are attributable to the infection itself [235]. SSIs are associated with an estimated annual healthcare cost ranging from USD 3.5 billion to USD 10 billion in the United States [233].

Biofilms significantly contribute to the pathogenesis of SSIs, with more than 80% of these infections associated with biofilm-forming bacteria, such as *S. aureus* and *Staphylococcus epidermidis* [236]. Pathogens adhere to surgical implements, including suture materials, and form biofilms that protect them from immune responses and antimicrobial agents, thus worsening infections [237,238].

Ongoing research elucidates the connection between biofilms SSIs. Research on *Enterobacteriaceae* isolates SSIs has clarified the mechanisms underlying biofilm formation and antibiotic resistance, providing an understanding of how these pathogens evade treatment and persist in clinical environments [239]. The health complications associated with biofilms, ranging from persistent wounds to infections acquired in hospitals [Table 2].

## 7. Approaches to Prevent and Manage Infections Caused by Biofilms

Below are several ways to prevent and treat biofilm-associated infections, derived from the information presented.

### 7.1. Therapeutic Strategies

There are various strategies for managing biofilms through therapy, each with its own unique and innovative tactics. To effectively manage and prevent biofilms, it is crucial to understand the mechanisms behind biofilm resistance.

#### 7.1.1. DNase as a Biofilm-Disrupting Agent

Deoxyribonuclease (DNase) enzymatically degrades eDNA, a key structural component of the biofilm matrix. By breaking down eDNA, DNase disrupts the integrity of the biofilm, weakening its structure and making it more susceptible to external interventions. This degradation enhances the penetration of antibiotics into the biofilm, thereby increasing their efficacy and improving treatment outcomes for persistent infections [246,247].

DNase effectively treats biofilms in both in vitro and in vivo models. Studies indicate that DNase diminishes biofilm biomass and enhances the susceptibility of bacteria within biofilms to antibiotics [248]. In patients with cystic fibrosis, DNase therapy, specifically recombinant human DNase (rhDNase), is employed to degrade the DNA-rich mucus in the lungs [249]. This treatment indirectly interferes with biofilm-like aggregates, resulting in enhanced respiratory function. The capacity of DNase to improve antibiotic efficacy highlights its potential as a significant adjunct in therapies aimed at biofilms [250,251].

#### 7.1.2. RNAIII-Inhibiting Peptide (RIP) for Biofilm Inhibition

RNAIII-inhibiting peptide (RIP) has been identified as a potential therapeutic agent for inhibiting biofilm formation, especially in infections associated with *S. aureus*, including *MRSA* [252]. RIP operates by interfering with the QS system, a bacterial communication process that governs the expression of virulence factors and the formation of biofilms. RIP specifically targets the RNAIII activating protein (RAP), thereby inhibiting its phosphorylation of downstream molecules crucial for biofilm formation and the regulation of virulence [253,254].

RIP’s primary mechanism of action involves disrupting the accessory gene regulator (agr) system, a vital QS pathway in *S. aureus*. The agr system regulates RNAIII expression, a crucial effector molecule that upregulates adhesion proteins and other virulence factors essential for biofilm formation. RIP suppresses the production of these factors by inhibiting RNAIII activation, which impairs bacterial adhesion, biofilm maturation, and virulence. This disruption inhibits biofilm formation and destabilizes biofilms, making bacterial communities susceptible to immune responses and antimicrobial agents [255,256].

Research has demonstrated the efficacy of RIP in diminishing biofilm formation in both in vitro and in vivo contexts. In catheter-implanted animal models, Oliveira et al. (2021) demonstrated that RIP injections significantly reduced biofilm production and decreased infection rates [256]. The findings underscore RIP’s potential as a preventive approach for managing medical device-related infections, particularly in the context of biofilm formation, which poses a considerable challenge.

#### 7.1.3. DNABII Proteins

DNABII proteins, such as integration host factor (IHF) and histone-like protein HU, are essential for creating and preserving biofilms. These proteins attach to eDNA in the biofilm matrix, enhancing its stability and fortifying its structure [112]. Given their critical role in maintaining biofilm integrity, DNABII proteins provide a promising target for therapeutic approaches designed to disrupt biofilms and improve therapies for infections linked with biofilms.

DNABII proteins attach to eDNA inside the EPS matrix, inducing structural bending and organization that reinforce the biofilm’s three-dimensional architecture [112]. This stability improves the biofilm’s resistance to physical disturbances, immunological responses, and antimicrobial substances. The ubiquitous existence of DNABII proteins across bacterial species renders them a universal and broad-spectrum target for biofilm removal [257].

Anti-DNABII antibodies provide a viable therapeutic strategy for breaking the connection between DNABII proteins and eDNA, weakening the biofilm matrix. These antibodies specifically target the DNA-binding domain of DNABII proteins, compromising the structural integrity of the biofilm and rendering bacterial cells susceptible to immune responses and antimicrobial treatments [258,259]. For instance, Martyn et al. revealed that antibodies targeting IHF impeded biofilm development on sinus implants, underscoring their prospective use in addressing biofilm-associated infections in clinical environments [260]. Devaraj et al. similarly observed that anti-DNABII antibodies interfered with biofilms produced by many human pathogens, demonstrating their extensive potential in addressing biofilm-related diseases [261].

#### 7.1.4. Bacteriophages

Phages, also known as bacteriophages, contain enzymes crucial for breaking down the EPS found in biofilms. This allows them to penetrate and modify the structure of the biofilm. The enzymes VAPGHs, endolysins, and depolymerases are recognized as powerful agents in phages’ ability to combat biofilms [262]. Tian et al. (2021) have highlighted the potential of phages to produce enzymes that break down the polysaccharide matrix and proteins in biofilms, offering an alternative approach to controlling bacterial biofilms [263]. Studies have shown that bacteriophage depolymerases facilitate the infiltration of phages into biofilms by breaking down the EPS substrate, ultimately leading to the destruction of bacterial cells [264]. Furthermore, it has been discovered that phages equipped with virion-associated depolymerases can overcome the challenge of biofilm matrix viscosity, allowing them to penetrate the biofilm and destroy EPS [265]. In addition, some phages have been found to prompt their hosts to produce EPS-degrading enzymes, which are subsequently released during phage replication to enhance the penetration of virions into the biofilm [266]. This mechanism highlights the diverse tactics employed by phages to overcome the challenges presented by biofilms.

Recent research has indicated that utilizing phage cocktails, which are blends of phages, is a superior approach to combatting harmful bacterial biofilms compared to using individual phages [267]. However, it is crucial to recognize that creating phage cocktails requires careful consideration, as the selection and assessment of phage combinations are critical in avoiding any unforeseen negative interactions within the cocktail. These interactions could trigger bacterial stress responses or encourage biofilm formation [268].

Integrating phage treatment and antibiotics represents a significant advancement in this field. Research has revealed that the combined use of phages and antibiotics, including ciprofloxacin, significantly enhances the elimination of biofilms [269,270]. The synergistic application of phage cocktails in tandem with antibiotics has demonstrated a marked improvement in reducing mature biofilms compared to using phages or antibiotics alone. This multi-dimensional approach highlights the evolving nature of biofilm treatment, leveraging the benefits of both phages and traditional antibiotics.

### 7.2. Antimicrobial Peptides

Antimicrobial peptides (AMPs) use various methods to combat biofilms, primarily by destroying bacterial cell membranes. These peptides target different molecular components at various stages of biofilm development, employing unique modes of action. Studies have shown that AMPs can rupture bacterial cell membranes, as demonstrated by atomic force microscopy [271,272]. In addition, AMPs have been found to physically damage bacterial cell membranes, ultimately leading to cell death. As a result, they are being explored as potential and effective antibacterial agents against biofilms [273].

Effective delivery methods are critical to maximizing the therapeutic potential of AMPs against biofilm-embedded pathogens. Nanoparticle-based delivery systems, for instance, encapsulate AMPs to protect them from enzymatic degradation while enhancing their penetration into the biofilm matrix. This approach ensures localized action and minimizes off-target effects [274,275]. Hydrogel systems represent another promising method, enabling sustained and controlled AMP release directly at the infection site and maintaining therapeutic concentrations over extended periods [276]. Additionally, self-assembling AMP-based nanostructures have shown potential in targeting biofilms, as they improve the stability of peptides and enhance their ability to interact with and disrupt biofilm components. These delivery innovations significantly enhance AMP efficacy, making them more robust tools for managing biofilm-associated infections [277].

In addition, the effect of AMPs on the control of genes linked to the formation of biofilms is an essential aspect of their antibacterial function. Particular AMPs are demonstrated to significantly influence biofilm formation by regulating genetic pathways that suppress biofilm development. The gene *YWP1*, exclusive to the yeast form of specific fungi such as *C. albicans*, and the gene *NRG1*, which produces a repressor of the filamentation pathway, have been recognized as crucial regulators in this process. Both genes demonstrate heightened activity when exposed to AMPs, resulting in improved suppression of biofilm formation [278].

Furthermore, studies have shown that AMPs can impede biofilm production by disrupting intercellular communication in bacteria. This disruption of QS, the communication system bacteria utilize to coordinate biofilm creation, reduces the ability to generate these protective structures [279].

In addition to their direct attack on pathogens, AMPs play a crucial role in regulating the immune response, thereby increasing the body’s ability to combat illnesses associated with biofilms [271]. This dual ability of AMPs to directly attack microorganisms and enhance the immune response contributes significantly to their therapeutic effectiveness in treating infections linked to biofilms. Several peptides, including human-defensin 3 (hBD-3), sheep myeloid peptide (AMP-29), rat cathelin-related antimicrobial peptide (rCRAMP), and bovine myeloid antimicrobial peptide (BMAP-27), demonstrate potent microbicidal activity against pathogenic bacteria such as *E. coli*, *P. aeruginosa*, *MRSA*, and *A. baumannii*. These peptides also inhibit biofilm formation and regulate the host’s immune system [280,281]. Excitingly, the future development of AMPs as novel treatments involves creating multifunctional AMPs that integrate their direct antibacterial activity with additional functions like controlling the immune system response [282].

AMPs provide a powerful approach to targeting persister cells by disrupting bacterial membranes, bypassing conventional resistance mechanisms. Among recent developments, TM5, a broad-spectrum AMP, has shown notable efficacy in eliminating planktonic and persister cells in biofilms developed by Gram-positive and Gram-negative bacteria [283,284]. Similarly, SAAP-148, an acyldepsipeptide, has demonstrated remarkable activity against *MRSA* persisters in a prosthetic joint infection model [285].

While AMPs are highly effective against biofilm-associated infections, their prolonged use poses a potential risk for resistance development. Microbial adaptation mechanisms, such as changes in membrane composition, efflux pump activation, and proteolytic degradation of AMPs, can diminish their effectiveness over time [286,287]. This emerging resistance threatens to limit the therapeutic utility of AMPs, especially in persistent infections. Monitoring resistance patterns and developing strategies to counteract adaptation, such as designing AMPs with novel sequences, rotating therapies, or combining AMPs with other antimicrobial agents, is essential to preserve their clinical relevance. Innovative approaches focusing on structural modifications to reduce susceptibility to resistance mechanisms further underscore the need to manage AMP-based therapies carefully [288].

### 7.3. Nanoparticle Strategies

Nanoparticles (NPs) have emerged as an innovative solution for combating biofilm-associated diseases due to their unique physicochemical characteristics, including diminutive size, extensive surface area, and potential for functionalization. These characteristics allow NPs to infiltrate biofilms’ EPS matrix, specifically target entrenched bacterial populations, and administer therapeutic medicines with exceptional accuracy [289,290]. Due to their considerable resistance to traditional antibiotics, biofilms are challenging; hence, nanoparticle-based approaches provide a viable option for preventing biofilm development and treating existing biofilm-associated illnesses [291,292]. Furthermore, integrating NPs with existing biofilm-targeting agents enhances their efficacy by improving penetration, stability, and targeted delivery within biofilms. This synergistic approach disrupts biofilm integrity and amplifies the therapeutic impact of antibiotics, antimicrobial peptides, and other agents, offering a highly effective strategy for managing persistent biofilm-related infections [293,294].

Topical formulations using NPs in chronic wound treatment break biofilms, reduce bacterial loads and enhance wound healing [291]. In dental treatment, NPs are used in adhesives and filling materials to impede biofilm growth, avoiding tooth cavities and periodontitis [295].

NPs interfere with biofilms via many methods. A primary mechanism is their capacity to infiltrate the thick EPS matrix, which often obstructs conventional antimicrobials. By permeating the matrix, nanoparticles provide therapeutic medicines directly to bacterial cells [296]. Metallic NPs, particularly silver (AgNPs) and zinc oxide (ZnO) possess inherent antibacterial characteristics, notably the generation of reactive oxygen species (ROS). These ROS damage the EPS matrix, destroy bacterial cell membranes, and impede metabolic processes, disintegrating biofilms [297,298]. Similarly, gold nanoparticles (AuNPs) have been identified to target major metabolic pathways: protein and nucleic acid synthesis, energy metabolism, and QS. These multifaceted mechanisms enable AuNPs to disrupt bacterial metabolism and communication, effectively impairing biofilm formation and persistence. Together, these metallic NPs offer promising strategies for managing biofilm-associated infections by targeting biofilms’ structural integrity and metabolic functions [299].

Metallic NPs, such as AgNPs, AuNPs, ZnO, and titanium dioxide, are thoroughly investigated for their significant antibacterial properties. Khan et al. (2019) showed that fucoidan-stabilized AuNPs significantly prevented biofilm formation and decreased pathogenicity in *P. aeruginosa* [300]. Gong et al. (2024) demonstrated that 3-amino-1,2,4-triazole-5-thiol-modified gold nanoclusters exhibit exceptional efficacy in eradicating biofilm-encased bacteria. Using an in vivo infected catheter implantation model, the study revealed that these gold nanoclusters could eliminate approximately 90% of the bacteria within the biofilms [301].

NPs may be engineered as vehicles for antimicrobial medicines, facilitating the targeted delivery of elevated medication concentrations directly to biofilms. This focused strategy minimizes systemic adverse effects while maintaining adequate medication levels at the infection site. Functionalizing particular ligands or peptides promotes accuracy by enabling selective targeting of bacterial biofilms, hence increasing treatment effects [302,303]. For instance, polymeric NPs, such as those composed of chitosan or poly (lactic-co-glycolic acid) (PLGA), provide enhanced benefits as carriers for antimicrobial compounds. Chitosan nanoparticles compromise bacterial cell walls and enhance medication delivery in biofilms. PLGA nanoparticles provide regulated drug release, guaranteeing prolonged antibacterial efficacy [304,305]. Nouruzi’s research (2023) demonstrated that PLGA NPs enhanced vancomycin penetration into *S. aureus* biofilms, markedly enhancing its efficiency [306]. Furthermore, Usnic acid (UA)-loaded chitosan (CS) NPs represent a promising strategy for combating infections caused by biofilm-forming pathogenic bacteria. The anti-persister activity of UA-CS NPs offers a targeted approach to addressing the challenges posed by persister cells, which are often resistant to conventional treatments. This innovative delivery system enhances the efficacy of UA by improving its bioavailability and penetration into the biofilm matrix, thereby disrupting the dormant bacterial population and potentially reducing biofilm-associated infections [307].

Lipid-based NPs, such as liposomes and solid lipid nanoparticles (SLNs), are increasingly recognized for their potential in biofilm control. Liposomes contain antibiotics, enhancing their penetration into biofilms, whereas functionalized liposomes specifically target bacteria associated with biofilms [308]. SLNs provide improved stability and extended release of encapsulated medicines, making them particularly beneficial in treating persistent biofilm-associated infections. Ferreira et al. have noticed that liposomes act as efficient nanoplatforms for improving the delivery of antibiotics into *S. aureus* biofilms. The integration of liposome-based delivery methods with the antibiotic vancomycin markedly suppressed *S. aureus* biofilms in their investigation. Encapsulating vancomycin in liposomes enhanced its diffusion across the biofilm’s EPS matrix, allowing elevated antibiotic concentrations to the bacterial cells situated inside the biofilm architecture [309]. This synergistic strategy underscores the capability of liposomes to address the difficulties presented by biofilm-related diseases, especially those involving resistant organisms like *MRSA*.

Despite their significant potential, several obstacles limit the widespread application of NPs in biofilm control. Among these, toxicity and biocompatibility are critical concerns. While NPs exhibit high efficacy against biofilms, ensuring they do not negatively impact human cells or induce inflammatory responses is paramount [292]. For instance, AgNPs, widely recognized for their antimicrobial properties, have been shown to trigger oxidative stress in human cells, which may lead to severe outcomes such as neurological diseases and genetic mutations [310]. Recent studies have further highlighted that exposure to AgNPs can cause cellular damage and increase the risk of neurological illnesses [310].

Additionally, investigations into the effects of AgNPs on human cells have revealed potential harm to genetic material and the induction of oxidative stress at certain concentration levels [310,311]. These findings also indicate genotoxic and cytotoxic effects associated with AgNP exposure, which underscores the need to evaluate their safety profiles carefully [312]. Addressing these challenges is essential to advance the clinical utility of NPs in biofilm management while minimizing potential risks to human health. Moreover, the scalability and cost-efficiency of nanoparticle manufacturing continue to pose substantial challenges. Producing NPs with uniform size, shape, and functionalization appropriate for therapeutic use necessitates sophisticated and expensive methods [313,314].

### 7.4. Antimicrobial Coatings

Antimicrobial coatings are crucial for reducing biofilm development in many settings, including medical equipment and healthcare environments. These coatings operate via two principal mechanisms: release-based and non-release techniques [315]. Coatings that release agents progressively discharge antimicrobial substances, like silver ions, antibiotics, or quaternary ammonium compounds (QACs). These compounds impede microbial growth upon contact, delivering a prolonged antimicrobial action that diminishes the probability of biofilm formation. The regulated release system guarantees prolonged effectiveness while reducing the need for frequent surface reapplication [316,317,318].

Conversely, non-release coatings inhibit microbial adherence by altering surface characteristics to resist attachment. These alterations often include hydrophobic, superhydrophobic, or zwitterionic surface designs that impede bacterial adhesion and hinder the early phases of biofilm development. Non-release coatings act as a passive but effective barrier against biofilm formation by focusing on the crucial adhesion phase [319,320,321].

Antimicrobial coatings are especially crucial in medical equipment, where biofilm-related infections pose substantial difficulties. Devices, including catheters, implants, and wound dressings, are susceptible to bacterial colonization, potentially leading to chronic infections and device malfunction. Silver-based coatings have shown effective in producing microbicidal surfaces that prevent pathogen adhesion and proliferation, diminishing biofilm development on urinary catheters and orthopedic implants [322,323].

Antibiotic-embedded polymer coatings provide targeted medication delivery, guaranteeing elevated concentrations of antimicrobial medicines at the possible infection site. This targeted administration improves therapy effectiveness while reducing systemic adverse effects. In addition to medical equipment, antimicrobial coatings are used on frequently touched surfaces in hospital environments, including bedrails and doorknobs [324]. These coatings successfully diminish contamination by pathogens such as *S. aureus* and *P. aeruginosa*, substantially decreasing the risk of nosocomial infections and the development of DSB [324,325].

Their durability and broad-spectrum antimicrobial activity, even against MDR, make these coatings highly efficient in maintaining hygienic conditions. By reducing the need for frequent cleaning and dependence on chemical disinfectants, these coatings enhance infection control and reduce maintenance costs and environmental impact. This combination of targeted efficacy, extended protection, and cost-effectiveness positions antimicrobial coatings as critical in combating biofilm-associated challenges in healthcare and other high-risk environments [326,327].

### 7.5. Antimicrobial Lock Solutions

Antimicrobial lock solutions (ALS) have become an essential approach for preventing and managing biofilm-related infections in catheters. ALS employs a targeted method by administering elevated doses of antimicrobial drugs directly into the catheter lumen, establishing conditions that deter bacteria colonization and biofilm development. This focused approach improves treatment effectiveness and markedly decreases catheter-related bloodstream infections [328,329]. ALS operates via many strategies to counteract biofilms. By introducing a concentrated antimicrobial solution into the catheter lumen [330,331], ALS infiltrates the biofilm matrix, disrupts the EPS, and exposes embedded bacteria to elevated concentrations of antimicrobial chemicals [332,333].

The composition of ALS is contingent upon the implicated pathogens and the clinical situation. Antibiotic-based ALS often uses compounds like vancomycin and daptomycin. These solutions are aimed against Gram-positive bacteria, including *S. aureus* and CoNS, which are prevalent in biofilm-related illnesses [334,335]. Also, caspofungin has proven effective against drug-resistant *C. auris* and Candida-bacterial polymicrobial biofilms during catheter-lock therapy when used in solutions with low ionic concentration [336].

Non-antibiotic ALS, including ethanol and taurolidine, provide alternate strategies that reduce the likelihood of antibiotic resistance. Ethanol interferes with microbial membranes and solubilizes biofilm matrix, while taurolidine has extensive antibacterial efficacy and prevents biofilm development [337,338]. Adjuvants such as ethylenediaminetetraacetic acid (EDTA) enhance the effectiveness of antibiotics by chelating vital metal ions that stabilize biofilms [339]. Combination therapy, such as minocycline–EDTA–ethanol solutions, has shown enhanced effectiveness in eliminating biofilms produced by *C. auris* [340].

Notwithstanding their benefits, ALS encounters certain obstacles, notably the risk of antimicrobial resistance linked to extended use of antibiotic-based treatments. While non-antibiotic ALS reduces this danger, it must be meticulously given to prevent systemic toxicity, particularly in ethanol-based formulations. Future developments in ALS formulations will likely emphasize hybrid strategies that integrate antibiotics with non-antibiotic compounds or use nanotechnology to enhance effectiveness and reduce side effects. An overview of strategies to prevent and manage biofilm-related infections is shown in [Table 3].

## 8. Challenges in Biofilm-Associated HAIs

### 8.1. Difficulty in Diagnosis and Detection

Diagnosing HAIs associated with biofilms poses considerable difficulties. Biofilms provide a protective matrix that conceals bacteria from standard detection techniques used for planktonic bacteria, often leading to false-negative outcomes [356]. Acquiring samples from medical devices or tissues containing biofilms complicates the process, as the complicated composition and integration of bacteria within the EPS matrix obstruct the identification of individual pathogens by standard culture methods [357]. Another significant diagnostic challenge is the existence of bacteria in a viable but non-culturable (VBNC) form inside biofilms. Bacteria persist in this form without proliferating on regular culture medium, complicating detection efforts [358]. This presents a considerable challenge to correctly identifying and treating biofilm-associated illnesses since conventional microbiological techniques may not detect bacteria in VBNC forms [359].

To address these diagnostic issues, several innovative methodologies have been investigated. Molecular approaches have shown efficacy in identifying biofilm-associated genes and virulence factors, including DNA sequencing, metagenomics, multiplex PCR, and metatranscriptomics [297]. Recent improvements in fiber-tip ball resonator optical fiber sensors and speckle analysis provide accurate capabilities for biofilm identification [360,361]. Nonetheless, despite their promise, the restricted accessibility of these technologies in healthcare environments continues to hinder wider implementation.

Overcoming the challenges of diagnosing biofilm-associated HAIs necessitates developing and adopting diagnostic tools that are more accessible, sensitive, and specialized. These innovations are essential for promptly and effectively managing biofilm-related infections, thereby alleviating their impact on healthcare systems. A key consideration in this process is implementing such advanced diagnostics’ economic and operational feasibility. Although initial equipment, staff training, and infrastructure investments may seem costly, their long-term advantages are substantial [362]. Enhanced biofilm detection enables more targeted antimicrobial therapy, reduces the recurrence of infections, and shortens hospital stays, ultimately offsetting the initial expenses.

Practical and cost-effective options, such as point-of-care biosensor technologies, hold particular promise, especially in resource-limited settings. These tools enable rapid and accurate biofilm detection, ensuring their vital role in clinical workflows and substantially improving patient outcomes [363]. Early detection of biofilm formation allows for timely intervention and prevents the progression of chronic infections, thereby reducing treatment complexity and associated healthcare burdens. Such advancements in diagnostic tools are pivotal for enhancing the precision and effectiveness of biofilm-associated HAI management, making them indispensable for modern clinical practices [364].

### 8.2. Biofilm Resistance to Disinfectants

A primary problem in controlling HAIs is biofilms’ inherent resistance to disinfectants. Biofilms provide a protective environment for bacteria, safeguarding them from the impacts of several disinfectants and antimicrobial therapies, thereby enabling pathogens to survive under rigorous infection control protocols [365]. This resistance is especially significant in DSBs, which develop on healthcare surfaces and have enhanced tolerance to disinfection and physical removal methods relative to wet surface biofilms and planktonic bacteria [366,367].

One such instance is *C. auris*, a multidrug-resistant fungus that has shown resilience to disinfectant doses often used in clinical environments, as documented by Kean et al. (2018). This capability complicates surface sterilizing and heightens the risk of HAIs linked to this fungus [191]. Likewise, biofilm-forming bacteria, including *A. baumannii*, exhibit resistance to commonly used disinfectants such as benzalkonium chloride and chlorhexidine, as emphasized in the study by Jiang et al. [368]. These results highlight the inadequacies of commonly used disinfectants in eliminating biofilms and controlling HAI-related bacteria.

Healthcare institutions have used many cleaning and disinfection methods to tackle these difficulties. Strategies include bleach, QACs detergents, ultraviolet irradiation, hydrogen peroxide vapor (HPV), and copper-treated surfaces or textiles [356,367]. HPV systems, in particular, have emerged as a powerful tool, capable of penetrating biofilm structures and eradicating embedded pathogens through the generation of ROS. These approaches have demonstrated efficacy in decreasing the occurrence of HAIs and MDROs [369,370]. Despite these measures, the enduring presence of biofilm-associated organisms underscores the need for ongoing innovation and enhancement in disinfection protocols.

Improving cleaning practices and investigating innovative disinfection technologies are essential to reduce biofilm-related hazards. Future research must concentrate on creating chemicals and techniques that may breach the protective biofilm matrix, thereby enhancing disinfection efficacy and decreasing the incidence of HAIs.

### 8.3. Economic Burden

The economic ramifications of HAIs associated with biofilms are significant, including direct hospital expenditures, supplementary costs, and broader social effects. Infections connected with biofilms often need extended hospitalizations, heightened antibiotic consumption, and supplementary medical procedures, substantially escalating healthcare costs [199]. In addition to direct medical expenses, these illnesses incur indirect costs such as diminished productivity and prolonged patient care, highlighting the financial significance of biofilm-associated HAIs.

Biofilm-associated MDROs exacerbate the financial burden, increasing morbidity, mortality, and healthcare expenditures. In the United States, HAIs result in roughly 88,000 deaths per year and incur an economic burden of USD 4.5 billion [5]. Similarly, a study conducted across hospitals in China identified pharmaceutical expenses, particularly antimicrobial drugs, as the primary contributor to the financial burden of HAIs, accounting for roughly 60% of the total economic impact [371].

Lost bed-days exacerbate the cost burden on healthcare systems. In the UK, HAIs are predicted to result in an annual loss of 58,010 bed-days, highlighting the inefficiencies and resource limitations associated with these illnesses [6]. These numbers demonstrate the extensive effect of HAIs on healthcare facilities, including heightened costs and diminished ability to serve other patients owing to extended hospital stays.

The substantial economic impact of biofilm-associated healthcare-associated infections underscores the pressing need for efficient preventive and control techniques. Investments in sophisticated diagnostic instruments, novel cleaning techniques, and tailored antibiotic treatments might diminish the prevalence of biofilm-associated illnesses, mitigating their economic burden on healthcare systems worldwide.

## 9. Biofilm Prevention and Control

Preventing and controlling biofilm-related HAIs requires a proactive and holistic strategy. The significant occurrence of biofilms in hospital environments and its correlation with MDROs in HAIs highlights the need for stringent infection control protocols [187]. Studies demonstrate that as much as 35% of HAIs may be averted by robust infection control initiatives and monitoring, highlighting the want for meticulously crafted techniques to reduce biofilm-associated hazards [372].

Compliance with hand hygiene measures is essential for avoiding the transmission of biofilm-associated infections. Healthcare professionals’ hands may serve as conduits for biofilm transmission, presenting a considerable danger to patients. Regrettably, healthcare professionals’ compliance rates with hand hygiene protocols persist low, fluctuating between 20 and 40% [373]. This figure underscores the need for extensive training and ongoing education for healthcare professionals about infection control practices. Advocating for rigorous compliance with hand hygiene protocols is fundamental to reducing biofilm development and related HAIs.

Effective disinfection and sterilization of medical instruments and ambient surfaces are essential for avoiding biofilm reservoirs and reducing the incidence of HAIs. DSBs provide a distinct problem due to their resistance to standard cleaning and disinfection methods and their ability to renew upon exposure to moisture and nutrients [374]. Efficient cleaning agents, including compounds with adamantane, have shown efficacy in reducing biofilm bulk and viable cell counts; nevertheless, their potential toxicity necessitates further assessment [375]. Consistent cleaning regimens specific to high-touch surfaces are essential since irregular cleaning prevents biofilm development [376,377].

Implementing antimicrobial stewardship programs is crucial for efficiently managing biofilm-associated HAIs. These initiatives aim to optimize antibiotic use to avert the development of resistance and decrease the occurrence of HAIs. Evidence-based antimicrobial stewardship programs promote appropriate antimicrobial use and assure conformity with local antibiotic susceptibility patterns [378,379]. Consistently revising hospital infection control policies to include antimicrobial stewardship may improve biofilm management initiatives.

Integrating modern surveillance and monitoring technologies is crucial for rapidly detecting and managing biofilm-associated HAIs. These systems assist healthcare institutions in detecting infection trends, identifying biofilm reservoirs, and executing targeted actions for their eradication. Recent innovations, including real-time automated nosocomial infection monitoring systems, have effectively decreased HAIs incidence by enabling early diagnosis and management [380,381] Surveillance facilitates outbreak management and enhances healthcare safety via ongoing monitoring and action.

An integrated approach incorporating personnel training, monitoring, and environmental sanitation is crucial for effectively tackling biofilm-related HAIs. Consistent training, rigorous hygiene protocols, and cutting-edge cleaning technologies can significantly reduce biofilm occurrence, enhance patient outcomes, and lower healthcare costs. By confronting the complex challenges biofilms pose head-on, healthcare institutions will strengthen their infection control measures and provide a safer environment for patients and staff.

## 10. Conclusions and Prospects for the Future

This thorough analysis has examined the complex interaction between biofilms and HAIs, a significant concern in contemporary healthcare. Biofilms are distinguished by their durability, flexibility, and ability to circumvent standard therapies, making detecting, managing, and preventing biofilm-associated HAIs a significant problem. This work elucidates the genetic and environmental variables influencing biofilm growth, offering critical insights into the processes behind their persistence and the related therapeutic implications. These results are essential for guiding focused initiatives to tackle this global health issue.

An important takeaway is the function of biofilms in promoting antibiotic resistance. This highlights the pressing need for sophisticated diagnostic methods and novel therapy strategies. Effective solutions must prioritize the prompt detection of biofilms with their prevention and treatment. This study emphasizes promising treatments such as bacteriophage therapy, QS inhibitors, and the creation of biofilm-targeted antimicrobial medicines. Moreover, robust antibiotic stewardship initiatives are crucial for reducing the development of resistance.

Proactive strategies to prevent biofilm-related HAIS are essential in hospital environments. These encompass the use of premium detergents, disinfectants, sterilization protocols, and biofilm-resistant substances. Improved surface coatings and compliance with rigorous hospital requirements are also essential. Furthermore, continuous training and education for healthcare personnel are essential to guarantee the uniform implementation of appropriate infection control protocols.

Outbreaks and epidemics associated with biofilms have considerable consequences for public health, highlighting the need for effective monitoring systems and prompt response tactics. Thorough public health education, with revised regulations and recommendations that include the most recent research and technology innovations, is crucial to reducing the risks associated with biofilm-related illnesses. Enhancing regulatory frameworks and public health initiatives is essential for successfully treating biofilm-related HAIs.

Future research should focus on creating innovative antimicrobial drugs and therapeutic approaches, especially targeting biofilms. A comprehensive knowledge of the molecular processes contributing to biofilm resistance and persistence will be crucial for developing effective therapies. Cutting-edge environmental sanitation technology, monitoring systems, and comprehensive training programs for healthcare staff should be instituted to improve hospital infection control practices.

In summary, biofilm-related HAIs significantly affect public health and patient management. Combating these illnesses requires a cooperative, interdisciplinary strategy amalgamating scientific innovation, therapeutic progress, and practical policy formulation. By implementing a proactive and cohesive approach, the healthcare sector may significantly advance in alleviating the burden of biofilm-related infections, boosting patient outcomes, and promoting public health. Despite ongoing hurdles, a resolute dedication to collaborative efforts is crucial for addressing biofilm-associated HAIs and their effects on global health.

## Figures and Tables

**Table 1:** Mechanisms and illustrations of biofilm resistance to antimicrobial agents.

Mechanism	Description	Examples	Citation
Physical Barrier of the Biofilm Matrix	EPS matrix in biofilms functions as a physical obstacle, hence diminishing the infiltration of antimicrobial agents.	EPS in biofilms of *P. aeruginosa*, *S. aureus*, and *S. mutans* restrict the penetration of antibiotics.	[115,122]
Diminished Rate of Growth and Metabolic Activity	Bacteria within biofilms frequently exhibit reduced growth rates and diminished metabolic activity, rendering them less vulnerable to antibiotics that specifically target rapidly proliferating cells.	Cells in biofilms of *S. epidermidis*, *K. pneumoniae*, and Mycobacterium TB exhibit slow growth and are resistant to many medications.	[12,125]
Genetic Recombination and Variation	Biofilms facilitate genetic diversity via DNA alterations, resulting in variations that could potentially exhibit greater resistance to therapies.	Genetic alterations in the biofilms of *A. baumannii* and *P. aeruginosa* contribute to the development of drug resistance.	[114,135]
Stress Responses and Adaptive Resistance	Bacteria in biofilms possess the ability to adjust and acquire resistance mechanisms as a reaction to environmental stressors, such as the existence of antibiotics.	Adaptive reaction to several stressors, such as physical pressure, hypochlorous acid, and exposure to antibiotics.	[137,139,168]
Persister Cells	Biofilms consist of ’persister cells’ that are in a dormant state and possess a high level of tolerance to antimicrobial agents. These cells have the ability to reestablish the population after undergoing treatment.	Survival of persister cells in biofilms of *C. albicans*, *E. faecalis*, and *S. aureus* following different treatments.	[12,156,157]

**Table 2:** An examination of the health complications associated with biofilms, ranging from persistent wounds to infections acquired in hospitals.

**Aspect**	**Description**	**Examples**	**Citation**
Chronic Wounds	Chronic biofilm infections hinder the process of healing. Biofilms provide protection for bacteria against therapies and immunological reactions. Result in extended inflammation and hinder the healing of wounds.	Conditions such as diabetic foot ulcers, venous leg ulcers, pressure ulcers, and surgical wound infections.	[209,210,211,212]
Medical Device Infections	Biofilms develop on medical equipment such as catheters and prostheses. Device-related infections can be induced, and their treatment can be challenging without the removal of the device. Possibility of dissemination of infection throughout the body.	Catheters, sutures, orthopedic implants, heart valves, and vascular grafts	[15,192]
Antibiotic Resistance	Biofilms enhance the transmission of genes that confer resistance. Offer resistance against antibiotics. The administration of elevated antibiotic doses is necessary for treatment, hence facilitating the emergence of antibiotic resistance.	*MRSA* present in biofilms, *P. aeruginosa* is commonly seen in lung infections associated with cystic fibrosis.	[114,115]
Dental Health	Dental plaque is a prevalent accumulation of microorganisms in the mouth. Results in dental caries, inflammation of the gums, and advanced gum disease. Controlled with consistent oral hygiene habits.	The conditions mentioned are dental caries (tooth decay), gingivitis, periodontitis, and dental implant-related mucositis.	[240,241]
Hospital-Acquired Infections	Hospitals pose a significant risk for infections caused by biofilms. Infection transmission is facilitated by the presence of biofilms on medical equipment, water systems, and hands. Challenge for individuals with immunodeficiency or intrusive medical devices.	Central line-associated bloodstream infections (CLABSI), ventilator-associated pneumonia (VAP), SSIs, and hospital-acquired urinary tract infections.	[242,243,244,245]

**Table 3:** Overview of strategies to prevent and manage biofilm-related infections.

Approach Category	Description	Examples	Citation
Therapeutic Strategies	Entails the utilization of precise pharmaceuticals and treatment procedures, frequently customized to counteract the tenacious characteristics of biofilms.	Combination antibiotic therapy, QS inhibitors, biofilm-dispersing medicines such as DNase, and RNAIII-inhibiting peptide (RIP) are also potential treatments and phage therapy.	[341,342,343,344]
Antimicrobial Peptides	Utilizes brief peptides with antibacterial characteristics.	Peptides such as nisin, colistin, cathelicidins, and defensins specifically target bacteria that produce biofilms.	[345,346,347]
Nanoparticle Strategies	The process entails utilizing nanoparticles to infiltrate biofilms and administer antibacterial substances.	Gold, silver and liposomal nanoparticles containing antibiotics, iron oxide nanoparticles, and quantum dots for precise medication delivery.	[299,348,349]
Antimicrobial Coatings	Surface antimicrobial application refers to the use of chemicals that inhibit the growth of biofilms.	Coatings of copper or silver, coatings of quaternary ammonium polymers, and coatings of titanium dioxide are applied to medical devices.	[350,351,352]
Antimicrobial Lock Solutions	Employed in medical devices to deliver a concentrated amount of antibacterial substances at the location susceptible to biofilm development.	Available lock solutions include ethanol, antibiotic, and taurolidine solutions.	[353,354,355]

## Data Availability

Not applicable.
